# Dual Biologic Therapy Induces Remission in Refractory Crohn’s Disease With Vedolizumab and Ustekinumab

**DOI:** 10.1093/crocol/otae080

**Published:** 2024-12-17

**Authors:** Syed Adeel Hassan, Courtney Perry, Patrick Carey, Durham Colohan, Mohamed Gebril Eltaher, Nabila Dawoud, Mahmoud Elkammar, Waqas Rasheed, Casie Mayne, Amy Stuffelbeam, Deborah Flomenhoft, Terrence A Barrett

**Affiliations:** Division of Digestive Diseases and Nutrition, Department of Medicine, University of Kentucky College of Medicine, Lexington, KY, USA; Division of Digestive Diseases and Nutrition, Department of Medicine, University of Kentucky College of Medicine, Lexington, KY, USA; Division of Digestive Diseases, Department of Medicine, University of Cincinnati College of Medicine, Cincinnati, OH, USA; Department of Internal Medicine, University of Kentucky College of Medicine-Northern Kentucky Campus, Highland Heights, KY, USA; Department of Imaging Physics, MD Anderson Cancer Center, The University of Texas, Houston, TX, USA; Department of Internal Medicine, Griffin Hospital, Derby, CT, USA; Department of Internal Medicine, University of Michigan, Ann Arbor, MI, USA; Department of Medicine, University of Kentucky College of Medicine, Lexington, KY, USA; Division of Digestive Diseases and Nutrition, Department of Medicine, University of Kentucky College of Medicine, Lexington, KY, USA; Division of Digestive Diseases and Nutrition, Department of Medicine, University of Kentucky College of Medicine, Lexington, KY, USA; Division of Digestive Diseases and Nutrition, Department of Medicine, University of Kentucky College of Medicine, Lexington, KY, USA; Division of Digestive Diseases and Nutrition, Department of Medicine, University of Kentucky College of Medicine, Lexington, KY, USA

**Keywords:** Crohn’s disease, refractory, ustekinumab, vedolizumab disease clearance

## Abstract

**Background:**

Despite advancements in the therapeutic armamentarium for Crohn’s disease (CD), biologic and small molecule monotherapies are associated with sub-optimal response and remission rates. Utilizing dual biologic therapy (DBT) holds the potential to increase efficacy in the treatment of refractory or partially responsive CD. Evidence pertaining to this strategy remains limited.

**Methods:**

We retrospectively examined refractory CD patients treated with a combination of ustekinumab and vedolizumab. Outcomes to DBT at week (wk) 52 were compared to monotherapy. The primary outcome constituted corticosteroid-free remission. Secondary outcomes included adverse events, infections, hospitalizations, surgeries, treatment persistence, and disease clearance.

**Results:**

Sixteen of 21 active refractory CD patients (76%) on DBT achieved disease remission at wk 52. Mucosal healing was observed in 38% (*n* = 6), biochemical remission in 25% (*n* = 4), and both clinical and biochemical remission in 38% (*n* = 6). Of these patients, 50% (*n* = 8) achieved corticosteroid-free remission. Three patients (37.5%) with corticosteroid-free remission achieved complete disease clearance. Paired median fecal calprotectin decreased from 508 to 118 µg/g (*P* < .0001). Paired C-reactive protein median decreased from 1.04 to 0.50 mg/dL (*P* < .0001). Median Harvey Bradshaw Index score reduced from 7 to 2 (*P* = .003). Endoscopic healing was achieved with a paired simple endoscopic score for CD decrease from 6 to 3 (*P* = .013). Corticosteroid dependency reduced from 17 to 8 patients discontinuing altogether. Patients still requiring corticosteroids experienced a decrease in average daily dose from 9 to 6 mg (*P* = .045). At wk 52, 5 patients (24%) did not meet the criteria for remission with 4 requiring CD-related surgical intervention. Mean CD-related hospitalizations reduced from 2.95 ± 2.33 to 0.52 ± 1.12 (*P* < .001) and surgeries from 1.76 ± 1.3 to 0.14 ± 0.4 (*P* < .001). Three infections with 1 requiring hospitalization and 1 report of headache were noted. Two patients discontinued DBT.

**Conclusions:**

Dual biologic therapy with ustekinumab and vedolizumab is a safe and effective strategy to induce disease remission in refractory CD. Large-scale studies are necessary to validate findings in a prospective setting.

## Introduction

Crohn’s disease (CD) is a chronic idiopathic subtype of inflammatory bowel disease (IBD) that presents with a remitting and relapsing clinical course.^1^ The pathognomonic hallmarks of CD include skip lesions involving any portion of the gastrointestinal tract with transmural inflammation.^1^ Results of unabated long-term inflammation include strictures, fistulas, abscesses, and colorectal cancer.^1^ In the United States, the incidence of IBD has substantially increased over the past several decades.^2^ An estimated 70 000 patients are diagnosed with new onset IBD annually.^2^ Overall approximations have yielded an increasing burden of IBD in adults with a prevalence of 2.4-2.7 million.^2^ Specifically, the incidence of CD is estimated at 4.1-20 cases per 100 000 population.^2,3^ Subsequently, IBD-related healthcare expenditures have risen annually by 7.1%.^4^

Historically, non-biologic therapies, including anti-inflammatory agents, immunosuppressors (thiopurines, 6-mercaptopurines, and methotrexate), and corticosteroids formed the cornerstone of CD therapeutic regimens.^5^ However their clinical utility was limited to symptom control with no impact on the underlying natural disease course.^5^ In 1997, the introduction of the first tumor necrosis factor inhibitor (TNFi), infliximab revolutionized the management of IBD.^6^ Advancements in experimental platforms accelerated the identification of molecular targets resulting in a diverse therapeutic armamentarium for CD. The technology enabled rapid incorporation of newer TNFi, anti-interleukins (ustekinumab, risankizumab), anti-integrins (natalizumab, vedolizumab), and small molecules including Janus kinase inhibitor (upadacitinib) in the management of moderate-severe CD.^5^ The subsequent ability to alter disease course shifted the intended treatment target from symptomatic control to well-defined clinical endpoints including mucosal healing, endoscopic healing, histologic healing, and biochemical marker normalization.^7^ This approach is often termed the “treat to target” approach.^7^

Despite significant advancements in medical management, 50% of CD patients undergo IBD-related bowel surgery within the first 10 years of diagnosis.^8^ Surgery is seldom curative with disease sequelae recurring in 80% of CD patients.^9^ Extra-intestinal manifestations (EIMs) further complicate care in 25%-40% of CD patients.^10^ In these subsets of patients, uncontrolled symptoms are reported despite adequate control over luminal disease. In the landmark UNITI and GEMINI clinical trials, disease remission was attained in 35%-60% of CD patients cumulatively.^11,12^ A pooled analysis of 25 major CD randomized controlled trials (RCTs) of biologic monotherapies across all classes from 1997 to 2022 revealed that the clinical remission and response rates in induction and maintenance phases have plateaued over time.^13^ Therapeutic response rates vary and remain suboptimal across drug classes. Subsequently, biologic-experienced patients experience diminishing response rates with each subsequent change in therapy.^14^ These results led experts to propose that drug selection, mono-therapeutic strategies, and sequencing result in a therapeutic ceiling limiting clinical benefit.

In order to overcome this therapeutic plateau and improve response rates, combining biologics to target multiple immune-physiological pathways has been trialed on an experimental and off-label basis. Current treatment guidelines do not provide recommendations regarding the use of dual biological therapy (DBT).^15^ Its use has been proposed in patients with uncontrolled EIMs with controlled luminal disease and refractory CD.^16^ Given the encouraging individual safety and efficacy profiles of vedolizumab (VDZ) and ustekinumab (UST), combining both therapies presents a unique opportunity for patients with refractory disease to achieve remission despite previous therapeutic failures. Herein, we assess the efficacy and safety of DBT with UST and VDZ. Its effect on steroid usage, surgical intervention, healthcare utilization, and treatment persistence was ascertained.

## Methods

### Study Design

The study adheres to the Strengthening the Reporting of Observational Studies in Epidemiology (STROBE) guidelines.^17^ This is a single-center retrospective observational analysis of outcomes pertaining to adult refractory CD patients identified in our IBD clinic over a 6-year period between January 1, 2018, and January 1, 2024. Our study diagnosis of CD was retrospectively cross-verified based on standard clinical, endoscopic, histologic, and radiographic criteria. Inclusion and exclusion criteria were set to optimally reflect the selection of patients to ascertain a snapshot of clinical outcomes and healthcare utilization on monotherapy at baseline versus on DBT at week (wk) 52. Patients were included if: (1) were above the age of 18, (2) deemed to have refractory CD based on clinicians’ assessment, (3) failed multiple therapies, (4) demonstrated partial response to monotherapy with UST or VDZ or received UST or VDZ as an additional therapy, (5) availability of evaluation data from the visit of DBT decision making (baseline), and (6) returned for follow-up at wk 52. We excluded CD patients if they: (1) were below the age of 18, (2) were on other concomitant biologic or immunomodulator therapies (except steroids), (3) were not deemed to have refractory disease, (4) did not receive either UST or VDZ as the second add on biologic therapy, and (5) failed to return for follow-up at wk 52.

**Figure 1.**
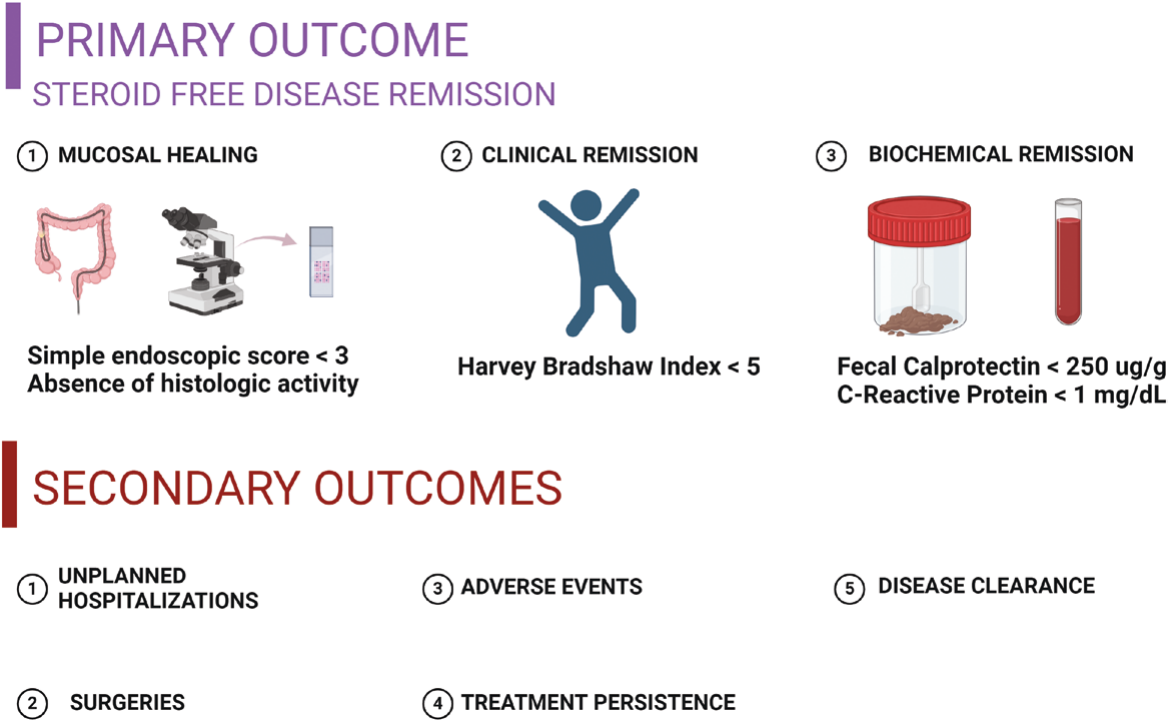
Summary of primary and secondary outcome measures.

For each eligible patient, pertinent data collection included demographics, medication history, surgical history, disease duration, type of steroid use, steroid dosage, hospitalizations, surgeries, infections, adverse events, and clinical characteristics such as endoscopic findings, histology, fecal calprotectin (FC), C-reactive protein (CRP), albumin, hemoglobin (HB), body mass index (BMI), and extra-intestinal manifestations (EIM) (Supplementary Figure 1). Crohn’s disease phenotype was classified using the Montreal classification. The primary outcome was steroid-free remission which was defined as one or more of the following: (1) mucosal healing, (2) biochemical remission, and (3) clinical remission. Secondary outcomes include adverse events, infections, CD-related hospitalizations, disease-related surgeries, treatment persistence, and disease clearance (Figure 1). When possible, transmural healing was analyzed by CT enterography (CTE). Readouts were collected at baseline when DBT was initiated. The date of the first induction infusion of the second agent in DBT was considered the starting time point for combination therapy. Subsequently, for each eligible patient, trends in disease status, steroid, and healthcare utilization were assessed at wk 52 and compared to baseline from the timepoint of initiation of DBT (post-DBT).

While a formal definition of refractory Crohn’s disease does not exist, it is regarded as the presence of persistent chronically active clinical, endoscopic, or histologic disease despite trials with multiple therapeutic modalities. All outcome measures were defined according to the “selecting therapeutic targets in inflammatory bowel disease” (STRIDE 2) guidelines.^7^ Clinical assessments were derived from the Harvey Bradshaw Index (HBI). Clinical response was defined as a change in HBI by ≥3 points. Clinical remission was defined as HBI <5. Mucosal healing was assessed utilizing a combination of simple endoscopic score (SES-CD) <3 and absence of histologic disease activity on surgical pathology. A >50% decrease in SES-CD was regarded as an endoscopic response. Biochemical remission was defined as normalization of FC <250 µg/g or CRP <1 mg/dL. Biochemical improvement was defined as a 50% reduction in FC or CRP from baseline. Trends in albumin, hemoglobin, and BMI were ascertained using internationalized standards. Clinical and biochemical remission parameters were used when endoscopic and histologic readouts were unavailable. In addition, as a secondary outcome, disease clearance was defined as the simultaneous achievement of clinical remission, endoscopic remission, histologic remission, transmural remission, and biochemical remission. Computed tomography enterography (CTE) was utilized to assess transmural remission (healing). Transmural remission was defined as bowel wall thickness (BWT) <3 mm and absence of hyperenhancement, comb sign, active inflammation, peri-enteric inflammation, abscess, fistulas, and strictures. The presence of any one of these radiographic features ruled out the possibility of transmural healing. Paired pre- and post-DBT assessments of CTE were utilized to assess transmural remission at wk 52. Patients with a stoma at baseline were not included in the analysis for clinical response/remission using HBI. Any patient with subsequent bowel surgery resulting in a stoma during the study period was only followed until the event. Frequencies and proportions of normalization and improvements in all variables were noted. The patient’s biochemical, endoscopic, and clinical profiles were regarded as normal only if the aforementioned criteria were met. A maximum therapeutic ceiling was defined as patients receiving 90 mg UST q4 wks and VDZ 300 mg q4 wks. The requirement for dose optimization of either VDZ or UST was assessed only on the basis of elevated FC, lack of clinical response, or evidence of disease progression on CTE. Treatment persistence was defined as the duration a patient remained on DBT from the time of treatment initiation till discontinuation or switching to another biological therapy. Discontinuation was defined as having a drug-free period greater than the dosing frequency of previous drug administration. Switching was defined as changing to a different medication prior to discontinuation. Overall rates, time to discontinuation, and reasoning were recorded at wks 52.

### Statistical Analysis

Frequencies and percentages were calculated for categorical variables. Continuous variables were reported as median and interquartile range (IQR). The normality assumptions for the distribution of continuous variables were assessed using the Shapiro-Wilk Test. Parametric variables following normal distribution were reported as mean ± standard deviation (SD). Comparison of paired non-parametric variables was performed using the Wilcoxon matched-pairs signed rank test to assess differences in CRP, FC, HBI, SES-CD, daily steroid dose, albumin, Hb, and BMI. Non-parametric variables following non-normal distribution were reported as median ± IQR. Comparison of paired parametric variables such as the number of hospitalizations and surgical events were performed using the paired *t*-test. A 2-tailed *P*-value ≤ 0.05 was set to be statistically significant. Statistical analyses were performed using GraphPad Prism 9.5.1 (GraphPad Software, Inc., San Diego, CA).

### Ethical Considerations

This study was conducted at the University of Kentucky after receiving appropriate approval from our university’s institutional review board (IRB approval #96010). The IRB granted exempt certification. All data security policies were implemented as per IRB policy. Informed consent for the study was waived by the IRB due to the inclusion of de-identified data only.

## Results

### General Characteristics

We identified 21 refractory CDs that received DBT with UST and VDZ only. The median age of patients in our cohort was 36 years (IQR: 23-47). The median duration of the disease was noted at 11 years (IQR: 6-21). Median age at diagnosis was 20 years (IQR: 15-27). Of the 21 patients, 12 were males (57%) and 9 were females (43%). The majority of patients had undergone prior CD-related intra-abdominal surgeries (81%, *n* = 17). The majority of our patients were current smokers (*n* = 13, 62%). Five patients were non-smokers (24%) and three were former smokers (14%). Stratification of disease phenotype as per Montreal classification revealed 38% (*n* = 8) were diagnosed before the age of 16 (A1), 52% (*n* = 11) were diagnosed between the ages of 17-40 years (A2), and 2 patients (10%) were diagnosed after the age of 40 (A3). The greatest proportion of patients had ileocolonic disease (L3) (*n* = 13, 62%), followed by colonic disease (L2) (*n* = 3, 14%), ileal disease (L1) (*n* = 2, 10%), ileocolonic and upper gastrointestinal (GI) (*n* = 1, 5%), colonic and upper GI (*n* = 1, 5%), and Ileal and upper GI (*n* = 1, 5%) (Table 1). The most common disease phenotype encountered was stricturing and penetrating (B2/B3) (*n* = 9, 43%), non-stricturing and non-penetrating (B1) (*n* = 5, 24%), penetrating disease (B3) (*n* = 4, 19%), and stricturing disease (B2) (*n* = 3, 14%) (Table 1). Ten patients (48%) had prior perianal involvement. None of the patients had active or sequelae of perianal disease. Extra-intestinal manifestations (EIM) were noted in 8 patients (38%). The most commonly observed EIM in our cohort was arthritis (*n* = 4, 50%) followed by aphthous ulcers (*n* = 2, 25%), hiradenitis suppurativa (*n* = 1, 12.5%), and primary sclerosing cholangitis (PSC) (*n* = 1, 12.5%).

**Table 1.** Summary of demographics and disease characteristics.

Variable	*n* (% or Interquartile range)
Gender	
Females	9/21 (43%)
Males	12/21 (57%)
Median age of cohort (IQR)	36 y (23-47)
Median disease duration (IQR)	11 y (6-21)
Tobacco history	
Smoker (*n*)	13 (62%)
Non-smoker (*n*)	5 (24%)
Former smoker (*n*)	3 (14%)
Median age at diagnosis (IQR)	20 y (15-27)
A1 (≤16 y)	8 (38%)
A2 (17-40 y)	11(52%)
A3 (>40 y)	2 (10%)
Disease location	
Ileal (L1)	2 (10%)
Colonic (L2)	3 (14%)
Ileocolonic (L3)	13 (62%)
Ileocolonic + Upper GI (L3 + L4)	1 (5%)
Colonic + Upper GI (L2 + L4)	1 (5%)
Ileal + Upper GI (L1 + L4)	1 (5%)
Disease behavior	
Non-stricturing, non-penetrating (B1)	5 (24%)
Stricturing (B2)	3 (14%)
Penetrating (B3)	4 (19%)
Stricturing and penetrating (B2 + B3)	9 (43%)
Perianal involvement	10/21 (48%)
Extra-intestinal manifestations	8/21 (38%)
Arthritis	4 (50%)
Oral aphthous ulcers	2 (25%)
Hiradenitis suppurativa	1 (12.5%)
Primary sclerosing cholangitis	1 (12.5%)
IBD-related Bowel surgeries	17/21 (81%)
Median failed therapies (IQR)	6 (4-7)
Median duration on first biologic (IQR)	22 months (12.5-32)
Prior anti-TNF use	19/21 (90%)
Prior thiopurine use	19/21 (90%)
Prior immunomodulator use	13/21 (62%)
Baseline steroid dependence	17/21 (81%)
None	4 (19%)
Prednisone	7 (33%)
Budesonide	10 (48%)

### Pre-DBT (Baseline)

**Figure 2.**
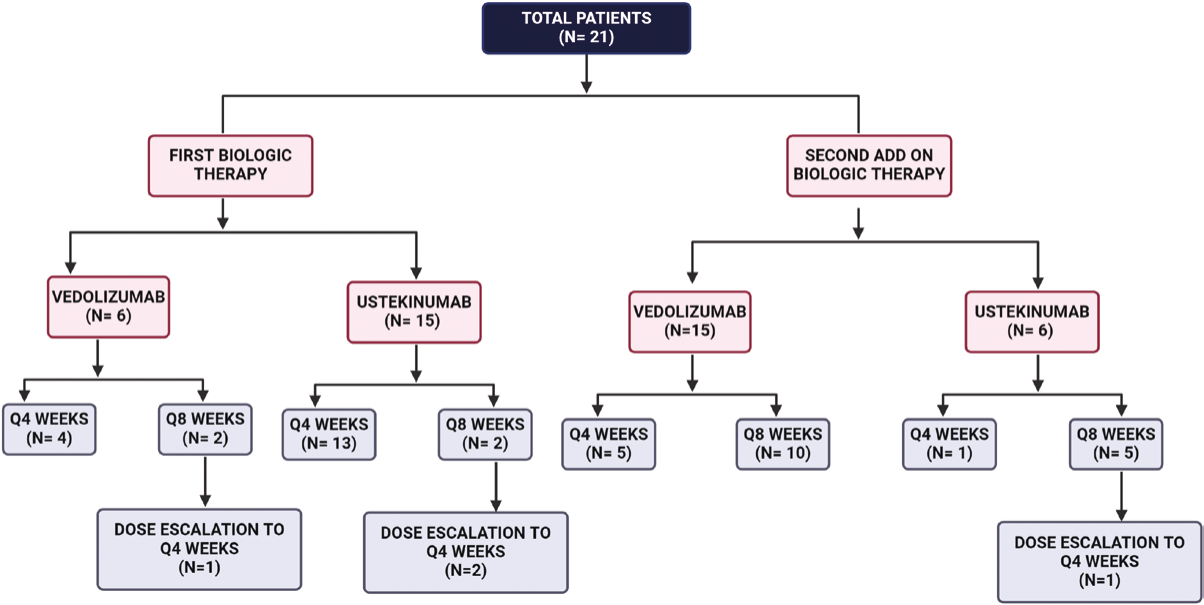
Distribution of therapeutic subsets based on biologic sequencing, frequency of administration, and dose escalation.

Twenty-one refractory CD patients (100%) had active disease despite monotherapy with either UST or VDZ. Fifteen patients (71%) were initially on UST monotherapy with 13 patients on q4 wk (87%) and 2 on q8 wk (13%) dosing. Six patients (29%) were on VDZ monotherapy with 4 on q4 wk (67%) and 2 on q8 wk (33%) dosing. All patients (*n* = 21, 100%) demonstrated a partial response to UST or VDZ monotherapy at baseline. Regardless of baseline biologic, the median time on the first mono-biologic therapy was 22 months (IQR: 12.5-32). Seventeen patients (81%) achieved the maximum therapeutic ceiling (eg, drug levels, dosing frequency) for either UST or VDZ mono-biologic therapy. As part of the standard of care, all patients were educated and offered combined VDZ or UST as a second alternate biologic. All patients (*n* = 21) approved and preferred the addition of a second biologic instead of a class switch or discontinuation. From the subset receiving VDZ (*n* = 6, 29%) as baseline monotherapy, UST was added as a second biologic with 1 patient on q4 wk and 5 patients on q8 wk dosing. In 16 patients receiving UST as baseline monotherapy, VDZ was added as a second biologic therapy with 5 patients on q4 wk and 10 patients on q8 wk dosing (Figure 2).

The most common clinical indications warranting the addition of UST or VDZ as a second biologic was active disease on colonoscopy (*n* = 11, 52%). Others include persistent EIMs (*n* = 8, 38%), elevated FC (*n* = 6, 29%), persistence of severe clinical symptoms (*n* = 3, 14%), and low albumin (*n* = 1, 4.7%). None of the patients had an end ileostomy or colostomy at baseline. Patients in our cohort failed a median of 6 advanced therapies (IQR: 4-7) including infliximab, adalimumab, methotrexate, and thiopurines. Significant baseline disease burden was noted with median HBI 7 (*n* = 21, IQR: 2-11), FC of 508 µg/g (IQR: 236.5-844, *n* = 17), CRP of 1.04 mg/dL (IQR: 0.49-2.9, *n* = 21), hemoglobin of 12.85 g/dL (IQR: 11.2-14.1 g/dL, *n* = 20), albumin 3.45 g/dL (IQR: 3-3.97 g/dL, *n* = 20), and BMI 27.2 (IQR: 20.15-34.55, *n* = 21). Eleven patients had baseline endoscopic assessments with a median SES-CD score of 6 (IQR: 3-7). Corticosteroid dependence was noted in 17 patients (81%). Budesonide (*n* = 10, 59%) was the most commonly utilized corticosteroid followed by prednisone (*n* = 7, 41%) (Table 1). Likewise, the median daily corticosteroid dose was 9 mg (IQR: 9-20). Increased burden of healthcare utilization on either UST or VDZ monotherapy was noted with a mean of 3 ± 2.33 CD-related hospitalizations and 2 ± 1.33 CD-related surgical events. Prior failed therapies include TNFi agents (*n* = 19, 90%), thiopurines (*n* = 19, 90%), and immunomodulators (*n* = 13, 62%). All nineteen patients with prior use of TNFi (*n* = 90%) experienced secondary loss of therapeutic response. All patients (*n* = 21, 100%) receiving DBT were naïve to either therapy with no indications of past treatment failure for either drug noted in medical records. No concomitant TNFi, immunomodulators, or thiopurine therapy was noted at baseline. At the time of DBT decision-making, CTE readouts were available for 17 patients (Supplementary Table 1) which indicated radiographic sequelae of persistent active disease with bowel wall thickness >3 mm, hyperenhancement, and active inflammation in 17 patients (100%). Other radiographic sequelae include peri-enteric inflammation in 14 patients (82%), comb’s sign in 9 patients (53%), fistulas in 4 patients (24%), and strictures in 4 patients (24%).

### Response to Dual Biologic Therapies

In terms of frequencies and proportions of normalization and improvements of various assessment readouts (Table 2), clinical response was noted in 20 of 21 patients (95%) with 1 patient exhibiting a lack of clinical response. The median time to response was 16 wks (IQR: 11.25-19.5). Fecal calprotectin normalized in 13 of 17 patients (76%), CRP normalized in 16 of 21 patients (76%), and HBI normalized in 16 of 21 (76%). When compared to DBT initiation, marked biochemical improvement was noted in 4 of 17 patients (24%) of patients for FC and 4 of 21 patients (19%) for CRP. Endoscopic response was noted in 4 of 11 patients (36%) (Table 2). Furthermore, 1 patient experienced endoscopic worsening with a 2-point increase in SES-CD from baseline.

**Table 2.** Reported rates of normalization and improvements in biomarkers, clinical scores, and endoscopic assessments at wks 52.

Variable	*n* (%)
Median time to response (IQR)	16 wks (IQR: 11.25-19.5)
Clinical response	20/21 (95%)
Non-response	1/21 (5%)
C-reactive protein normalization	16/21 (76%)
C-reactive protein improvement	4/21 (19%)
Fecal calprotectin normalization	13/17 (76%)
Fecal calprotectin improvement	4/17 (24%)
Harvey Bradshaw Index normalization	16/21 (76%)
Harvey Bradshaw Index worsening	5/21 (24%)
Endoscopic response	4/11 (36%)
Endoscopic remission	6/11 (55%)
Endoscopic worsening	1/11 (9%)
Mucosal healing	6/11 (55%)

Abbreviation: IQR, interquartile range.

Dose escalation of DBT was required in 4 patients (Figure 1). From the subset receiving monotherapy with VDZ q8 wks monotherapy at baseline, dose escalation to q4 wks was required in only 1 patient. Similarly, 2 patients received UST q8 wks as their initial biologic required dose escalation to q4 wks. Only one patient receiving UST q8 wks as add-on therapy required dose escalation to q4 wks. Clinical indications for dose escalation included persistently elevated FC (*n* = 2), lack of clinical response (*n* = 1), and evidence of disease progression on CTE (*n* = 1). Dose de-escalation was not observed in our cohort.

At wk 52, treatment with DBT persisted in 19 of 21 patients (90%). Overall, 2 patients discontinued therapy due to loss of insurance coverage and bowel resection at wks 48 and 51, respectively. The therapeutic plan for these patients was subsequently altered to dose escalation of VDZ q4 wk monotherapy and initiation of Risankizumab 360 mg q8 wks. None of the patients required a therapeutic switch in class or out of class during the 1 year follow-up period.

**Figure 3.**
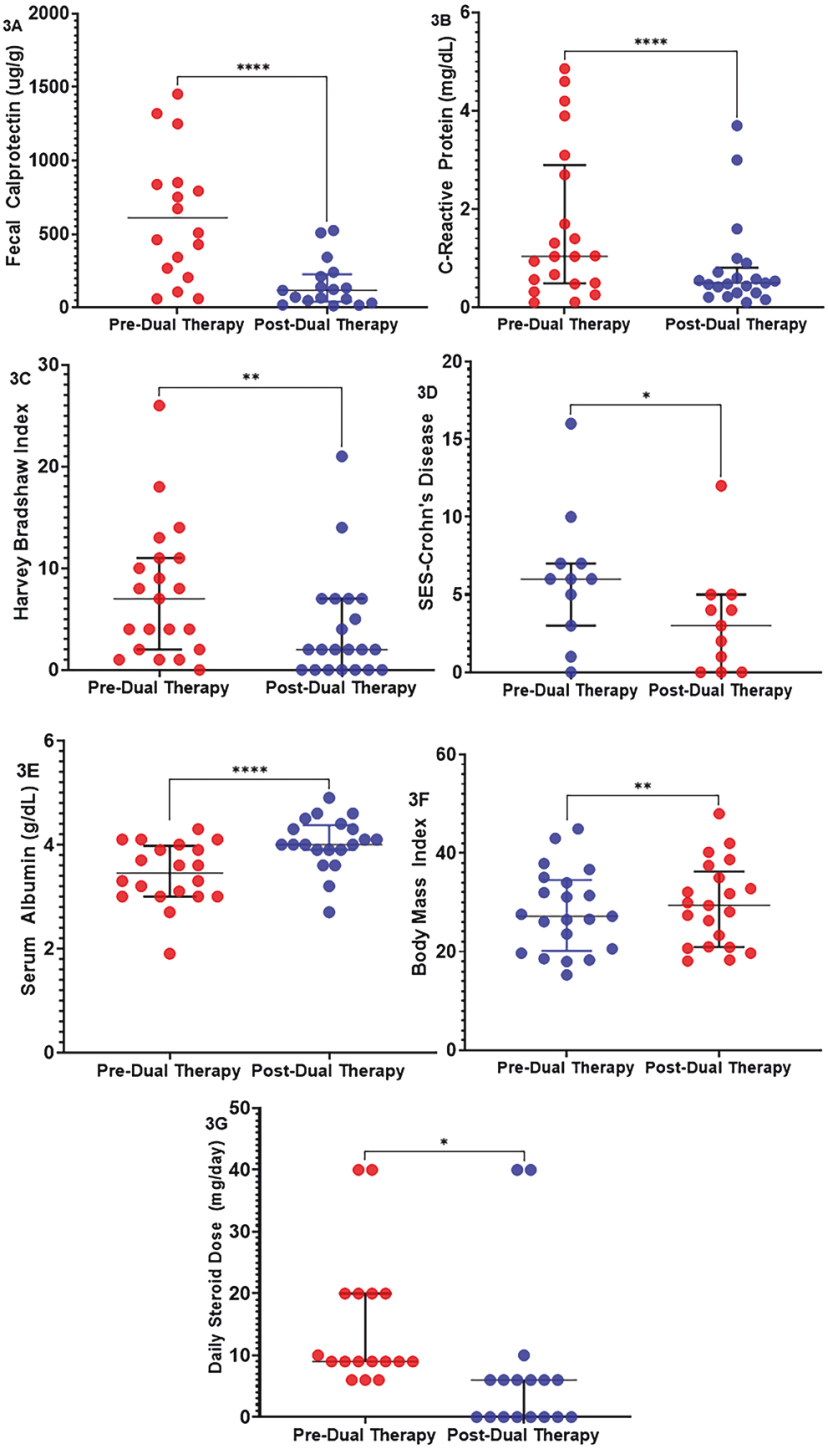
(A-G) Scatter dot-plot with median and interquartile range (IQR) showing the distribution of fecal calprotectin, c-reactive protein, Harvey Bradshaw index, simple endoscopic score, serum albumin, body mass index, and daily steroid dose for patients’ pre- and post-dual biologic therapy at wk 52, respectively.

The median FC in DBT patients decreased from 508 µg/g (IQR: 236.5-244) to 118 µg/g (IQR: 39.5-226, −74%, *P* < .0001) (Figure 3A, Table 3). The median CRP decreased significantly from 1.04 mg/dL (IQR: 0.49-2.9) to 0.50 mg/dL (IQR: 0.3-0.81, −52%, *P* < .0001) (Figure 3B). The median HBI decreased from 7 points (IQR: 2-11) to 2 points (IQR: 0-7, −47%, *n* = 21, *P* = .0031) (Figure 3C). The endoscopic disease burden also decreased with median SES-CD 3 (IQR: 0-5, −50%, *n* = 11, *P* = .013) (Figure 3D). Significant improvements in albumin were noted with the median increasing from 3.45 g/dL (IQR: 3-3.9) to 4 g/dL (IQR: 3.9-4.4, +17% *n* = 20, *P* < .0001) (Figure 3E). Median BMI increased by 4.5% from 27.2 to 29.4 (IQR: 20.95-36.25, *n* = 21, *P* = .0012) (Figure 3F). Although a modest increase in hemoglobin readouts was observed, this was statistically non-significant (*n* = 20; *P* = .39). In patients requiring corticosteroids, median daily dosages dropped from 9 mg (IQR: 9-20) to 6 mg (IQR: 0-6, −50%, *P* = .045, Figure 3G). Eight patients (47%, *n* = 17) discontinued corticosteroids and 2 required steroid dose escalation. EIMs resolved in 6 patients (75%, *n* = 8) with a resolution of enteropathic arthritis in 4 (67%, *n* = 6) and aphthous ulcers in 2 patients. There was no improvement in patients with an EIM of hidradenitis suppurativa and PSC. Paired CTE readouts were available for 11 patients with 6 patients demonstrating no sequelae of active disease and 5 demonstrating persistent active disease (Supplementary Table 2). Of the 6 patients demonstrating resolution of active disease on CTE, steroids were discontinued in 3 patients, and daily steroid dose was reduced in 3 patients. Only 3 had paired endoscopic and histologic disease assessments to evaluate for transmural remission and disease clearance. Pre- and post-DBT percentage changes of respective variables are demonstrated in Supplementary Figure 3.

**Table 3.** Pre- and Post-DBT trends in clinical scores, biochemical parameters, and healthcare utilization characteristics.

Variable	Pre-dual biologic therapy	Post-dual biologic therapy (wk 52)	*P* value
Median fecal calprotectin (IQR)	508 µg/g (236.5-244)	118 µg/g (39.5-226)	<.0001
Median C-reactive protein (IQR)	1.04 mg/dL (0.49-2.9)	0.50 mg/dL (0.3-0.81)	<.0001
Median Harvey Bradshaw Index	7 (2-11)	2 (0-7)	.0031
Median SES-CD	6 (3-7)	3 (0-5)	.013
Median albumin	3.45 g/dL (3-3.9)	4 g/dL (3.9-4.4)	<.0001
Median BMI	27.2 (20.15-34.55)	29.4 (20.95-36.25)	.0012
Median hemoglobin	13 (11.3-13.9)	13.65 (12.25-14.40)	.39
Median daily steroid dose	9 mg (9-20)	6 mg (0-6)	.045
Mean CD-related hospitalization (± standard deviation)	2.95 ± 2.33	0.52 ± 1.12	<.001
Mean CD-related surgeries (± standard deviation)	1.76 ± 1.3	0.14 ± 0.4	<.001

Abbreviations: BMI, body mass index; CD, Crohn’s disease; IQR, interquartile range; SES-CD, simple endoscopic score CD.

Based on our criteria for disease remission, 16 of 21 (76%) DBT patients achieved disease remission at wks 52 (Supplementary Figure 2). Specifically, mucosal healing was observed in 6 of 11 DBT patients (55%), biochemical remission in 4 of 16 (25%), and both clinical and biochemical remission in 6 of 16 patients (38%). Eight of 16 DBT patients (50%) achieved corticosteroid-free remission. Of the patients attaining steroid-free remission, 4 of 8 patients (50%) previously received budesonide, 2 of 8 (25%) received prednisone, and 2 patients (25%) never required any type of steroid introduction. Furthermore, 3 of 8 patients (37.5%) with corticosteroid-free remission achieved complete disease clearance with simultaneous achievement of endoscopic remission, histologic healing, transmural healing, clinical remission, and biochemical remission. When compared to the whole cohort, the rate of disease clearance was deemed at 14%. Additionally, radiographic sequelae of active disease resolved on CTE in In addition to discontinuation of steroids, these patients also showcased substantial reduction in healthcare utilization such as CD-related hospitalizations and surgeries.

Five patients (24%) did not meet the criteria for disease remission at wk 52. All 5 exhibited active features of disease sequelae on CTE. Four patients (80%) required surgeries for recurrent CD flares. One patient suffered a flare with worsening endoscopic disease (SES-CD = 12), an increase in HBI to 21, and required surgery with left extended hemi-colectomy of the diseased region. Post-surgery, this patient continued VDZ/UST dual therapy with budesonide 6 mg/day. This patient had a history of PSC. The remainder of 4 patients also exhibited persistent significant endoscopic and clinical disease burden with SES > 10 and HBI > 8. When compared to baseline endoscopic burden, 4 patients achieved endoscopic response. Of these, one patient required small bowel resection due to perforation. Furthermore, two patients required incision and drainage with fistulotomy and seton placement for recurrent perianal abscess and fistula-in-ano. Given the severity and chronicity of persistent disease, all 4 patients switched therapy to risankizumab after wk 52.

Treatment with UST/VDZ DBT reduced healthcare utilization with a drop in mean CD-related hospitalizations from 2.95 ± 2.33 to 0.52 ± 1.12 (−83%, *P* < .001) and surgeries from 1.76 ± 1.3 to 0.14 ± 0.4 (−93%, *P* < .001), respectively, (Table 3). After 1 year of VDZ/UST DBT, only 4 patients who did not attain disease remission required surgery. Six unplanned hospitalizations were noted in the cohort. Four hospitalizations were due to patients not meeting criteria for disease remission with 1 patient experiencing CD flare and 4 requiring surgeries (Table 4). One patient contracted community-acquired pneumonia (CAP) and required hospitalization. Two reported infections did not require hospitalizations. These include sinusitis and CAP managed with outpatient antibiotic therapy. One minor adverse event (headache) was reported. Total infection rates totaled 14% (*n* = 3). In our cohort, no serious infections were observed with the use of this therapeutic combination indicating favorable tolerability for severe refractory CD patients. All patients were compliant and retained VDZ/UST combination therapy with 2 patients discontinuing therapy post-surgical resection due to loss of insurance coverage (Table 4). Due to the small cohort size, predictors of response to UST/VDZ dual therapy could not be ascertained.

**Table 4.** Summary of adverse events and hospitalization.

Variable	*n* (%)
Hospitalizations	6/21 (29%)
CD flare	1
CD flare requiring surgery	4
Community acquired pneumonia	1
Not requiring hospitalization	3/21 (14%)
Sinusitis	1
Community acquired pneumonia	1
Headaches	1
Dual biologic discontinuation	2/21 (9.5%)
Post-surgical resection	1
Loss of insurance coverage	1

Abbreviation: CD, Crohn’s disease.

## Stratification of Results Based on Therapeutic Sequencing

### Addition of Ustekinumab as the Second Biologic

Six of 21 CD patients on VDZ monotherapy received UST as a second biologic. Dosing intervals were q8 wks in 5 patients and q4 wks in 1 patient. From the q8 dosing subgroup (*n* = 5), only 1 patient (20%) required dose escalation to q4 wks. The time to respond was 17 wks (IQR: 16-34.50). Paired FC readouts were available for 4 patients due to lack of compliance. Likewise, data for the assessment of endoscopic response was limited to 3 patients due to the lack of availability of paired SES-CD scores. Median FC reduced by 60.4% from 821.5 µg/g (IQR: 243-1203) to 325 µg/g (IQR: 85.5-521, *n* = 4). Median CRP reduced by 54% from 1.31 mg/dL (IQR: 0.99-2.65) to 0.60 mg/dL (IQR: 0.52-0.95). Median SES-CD reduced by 67% from 3 (IQR: 1-10) to 1(IQR: 0-12, *n* = 3). On the contrary, the median HBI increased by 25% from 4 (IQR: 1-9) to 5 (IQR: 0-14). Fecal calprotectin normalized in 50% of patients (*n* = 2/4), CRP normalized in 4 of 5 patients (80%), and HBI normalized in 2 of 5 patients (40%). Marked improvement in FC was noted in 2 patients with a 37% reduction from baseline FC. A simple endoscopic score for CD normalization with the absence of histologic disease was achieved in 2 patients. Additionally, at DBT initiation, 3 of 5 patients utilized transient corticosteroid therapy. Based on the type of corticosteroid use, prednisone and budesonide were utilized in 2 patients and 1 patient respectively. At wk 52, a 100% reduction in median daily corticosteroid dose (median: 0 mg; IQR: 0-10) was observed. Two of 3 patients discontinued corticosteroids altogether. Daily prednisone dose reduced by 50% (20-10 mg) in 1 patient.

Overall, 3 of 5 patients (60%) attained disease remission with mucosal healing in 2 patients, clinical remission, and biochemical remission in 1 patient. However, true steroid-free disease remission was observed in only 2 patients (40%). The addition of UST failed to provide any clinical benefit with DBT failure in the remaining 2 of 5 patients (40%). Reasons for therapeutic failure include worsening endoscopic and radiographic disease activity and persistent inflammatory and symptomatic burden at wk 52. Treatment with DBT significantly reduced mean CD-related hospitalizations by 81% (mean: 0.60 ± 0.89). The requirement for disease-related surgeries was reduced by 90% (mean: 0.20 ± 0.44). When stratified by the response, the greatest reduction in the burden of disease-related hospitalizations and surgeries was noted in responders. Treatment with DBT persisted through wk 52 in 4 of 6 patients (60%). Reasons for drug non-persistence include discontinuation post-bowel resection and loss of insurance coverage. No serious side effects to this particular DBT therapeutic sequencing were reported at wk 52.

### Addition of Vedolizumab as the Second Biologic

Sixteen of 21 CD patients on UST monotherapy received VDZ as a second biologic. Dosing intervals were q4 wks in 5 patients and q8 wks in 10 patients. Clinical response was achieved in all patients with a median time to response of 15.5 wks (IQR: 9-18). Paired FC levels were available for 13 patients. Likewise, data for the assessment of endoscopic response was limited to 7 patients due to the lack of availability of paired SES-CD scores. Median FC reduced by 84% from 462 µg/g (IQR: 236.5-794.5) to 72 µg/g (IQR: 24-173). Median CRP reduced by 46% from 0.86 mg/dL (IQR: 0.36-3) to 0.46 mg/dL (IQR: 0.24-0.67). Median SES-CD reduced by 50% from 6 (IQR: 5-7) to 3 (IQR: 0-5). Median HBI decreased by 75% from 8 (IQR: 2.5-12.5) to 2 (IQR: 0-6.25). FC normalized in 85% of patients (*n* = 11/13), CRP normalized in 13 of 16 patients (81%) and HBI normalized in 14 of 16 patients (88%). Marked improvement in FC was noted in 2 patients with a 28% reduction from baseline FC. SES-CD normalization with the absence of histologic disease was achieved in 4 patients (40%). Additionally, at DBT initiation, 14 of 16 patients utilized corticosteroids. At wk 52, a 33% reduction in median daily corticosteroid dose (median: 6 mg; IQR: 0-6) was observed. Based on the type of corticosteroid use, prednisone and budesonide were utilized in 5 patients and 9 patients respectively. Prednisone users experienced a 100% reduction in median daily dosage (median: 0 mg; IQR: 0-6). Patients on budesonide therapy experienced a 33% reduction in median daily dosage (median: 6 mg; IQR: 0-23). Overall, 6 of 14 patients discontinued corticosteroids altogether. Of the 8 patients continuing corticosteroids at wk 52, dose escalation was used in 2 patients (25%).

Overall, 13 of 16 patients (81%) attained disease remission with mucosal healing in 4 patients, clinical + biochemical remission in 5 patients, and only biochemical remission in 4 patients. However, true steroid-free disease remission was observed in 5 patients (31%). The addition of UST failed to provide any clinical benefit with DBT failure in the remaining 3 patients (19%). Reasons for therapeutic failure include worsening endoscopic disease activity and persistent symptomatic burden at wk 52. Treatment with DBT significantly reduced mean CD-related hospitalizations by 83% (mean: 0.50 ± 1.21). The requirement for disease-related surgeries was reduced by 92% (mean: 0.12 ± 0.34). When stratified by the response, the greatest reduction in the burden of disease-related hospitalizations and surgeries was noted in responders. In this subgroup of therapeutic sequencing, a 100% treatment persistence through wk 52 was noted. More therapy-associated adverse events were noted with the occurrence of pneumonia, sinusitis, and headaches in 1 patient each.

## Discussion

The clinical and symptomatic heterogeneity of CD is known to complicate patient management. This is attributed to varying disease location, disease behavior, age at disease onset, treatment status, and the presence of perianal disease and EIM.^18^ Modern transcriptomics have further delineated CD heterogeneity by yielding molecular signatures specific to disease location, behavior, and exposure to VDZ and TNFi therapy.^18^ These molecular signatures are also known to correlate with clinical outcomes.^19^ Furthermore, differentially expressed mucosal cytokines and chemokines specific to disease location and disease duration have been reported.^20,21^ These findings help establish the notion of heterogeneity in IBD pathogenic responses based on disease characteristics.

While patients can achieve symptomatic disease remission on corticosteroids, the myriad of associated adverse events limits its long-term utility.^22^ Data pertaining to the use of DBT in refractory CD remains limited. The advent of modern-day biologics and small molecules has diversified the mechanistic rationale in the treatment of IBD. Targeting multiple underlying therapeutic pathways by combining agents represents a sound strategy to help overcome heterogeneity in underlying IBD disease mechanisms. Combining therapies further provides the added benefit of improving overall efficacy synergistically while maintaining a safe profile.^23^ Most studies have assessed the utility of TNFi with a novel biologic. In 2007, the first-ever DBT study reported a well-tolerated safety profile and greater efficacy in CD patients receiving natalizumab plus infliximab versus infliximab alone.^24^ However, reports of increased incidence of progressive leukoencephalopathy secondary to JC virus infections led to its isolation in clinical care.^25^ To date 2 major RCTs have been conducted to assess the safety and efficacy of combination therapy in CD.^26,27^ Results from the SONIC Trial suggested that infliximab plus azathioprine combination therapy was superior in inducing remission when compared to patients who received azathioprine alone in moderate-severe CD.^26^ This combination of TNFi and immunomodulator is routinely used in clinical care to offset the development of immunogenicity to biologics.^26,27^ More recently, the EXPLORER trial assessed the utility of triple combination therapy with 2 concomitant biologics (VDZ, adalimumab) and oral methotrexate in moderate-severe CD.^27^ Results from the trial supported the safety and feasibility of combining therapies with up to 54% achieving clinical remission and 34% achieving endoscopic remission at wk 26.^27^ A post-hoc Bayesian analysis of different study arms from the EXPLORER trial further revealed a greater rate of endoscopic remission with triple combination therapy (33.5%) versus monotherapy with VDZ (27%) or adalimumab (30%).^27^ Favorable efficacy and safety of VDZ and UST monotherapy have been delineated in RCTs.^11,12^ Furthermore, lower rates of immunogenicity favor the use of VDZ/UST as a combination therapy.^28^ Currently in literature, most data pertains to the efficacy and safety of combining TNFi with a newer biologic. RCTs assessing the utility of combining UST and VDZ are yet to be conducted. However, results from case reports, case series, and retrospective studies are encouraging.^29–35^

Findings from our study have robustly added to a limited body of evidence. Our study represents one of the largest retrospective cohorts specifically analyzing the efficacy and safety of combination UST/VDZ to date. This is further backed up by findings of a recent high-profile meta-analysis where 288 trials of dual biologic or small molecule therapy were identified across 30 studies.^23^ The study revealed that the UST and VDZ combination accounted for only 54 out of the 288 reported patient combinations identified across 30 studies.^23^ This indicates the need for further prospective large-scale studies assessing the potential of this combination. The baseline disease severity in our patients is quite evident from our data. In our cohort, a median disease duration of 11 years and a median of 6 failed therapies was noted. Furthermore, concomitant stricturing and penetrating (B2/B3) phenotype and prior IBD-related surgeries in 81% confirm the severe baseline refractory nature of the disease. Therefore, our study is an encouraging assessment of DBT in high-risk refractory patients. Taken together, at baseline, our patients had a higher disease burden when compared with the only randomized controlled trial conducted to date.^26^

Despite smoldering disease, 16 (76%) patients achieved disease remission at wk 52. Specifically, mucosal healing was observed in 38% (*n* = 6), biochemical remission in 25% (*n* = 4), and both clinical and biochemical remission in 38% (*n* = 6). Of these patients, 50% (*n* = 8) achieved corticosteroid-free remission. We did not identify any new safety signals with minor manageable infections noted in 14%. At wk 52, significantly improved healthcare utilization in terms of hospital visits, unplanned surgeries, and a change in steroid utilization were noted when compared to baseline. Our overall achievement of remission rates is consistent with findings from previously published retrospective studies.^16,34^ Yang et al.^16^ reported 24 DBT trials in 22 patients with UST/VDZ combination accounting for 8 reported DBT combinations. Of the 8 patients, they reported 63% endoscopic improvement (5/8), 25% endoscopic remission (2/8), 71% clinical response (5/7), and 57% clinical remission (57%). In another study, from a total of 15 patients, 5 patients with UST/VDZ combination were reported.^34^ Overall, 4 achieved a clinical response, 1 required surgery, and 2 were able to reduce their corticosteroid.^34^ Glassner et al.^30^ conducted a retrospective study of 53 patients on various combination therapies. While results from the study failed to report outcomes in a combination-specific manner, 50% of patients achieved clinical, biochemical, and endoscopic remission.^30^ Llano et al.^32^ reported the utility of the VDZ/UST combination in 2 CD patients. Both patients achieved clinical remission with 1 adverse event of rotavirus.^32^ Privitera et al.^33^ reported achievement of clinical remission with no adverse events in 2 CD patients. Ninety percent of patients in our cohort were also TNFi-experienced. To stratify further, all patients who achieved objective measures qualifying them for remission were TNFi non-responders. This is quite significant as with each change of therapeutic class, the likelihood of attaining a clinical response to subsequent therapy reduces by 27% in previously TNFi-experienced patients.^14^ Our data shows that combining UST and VDZ can help overcome the phenomenon of sub-optimal remission and response rates in TNFi experienced patients.

Retrospective analysis of the steroid-free remission subgroup from our study revealed that 14% achieved complete disease clearance with simultaneous achievement of endoscopic remission, histologic remission, clinical remission, and biochemical remission. Overall, the rate of disease clearance was deemed at 14%. Disease clearance has been regarded as an emerging therapeutic outcome associated with superior disease control and reduced long-term disease complications.^36^ To date, only one prospective study conducted by Takenaka et al.^37^ has assessed the relationship between attaining disease clearance and long-term outcomes in CD patients. While some differences in the patient selection criteria exist, our results are consistent with improvement in long-term outcomes. In our cohort, reduced healthcare utilization was noted with decreased CD-related hospitalizations and surgeries in 83% and 93% of patients, respectively. Notably, the greatest reductions in healthcare utilization were noted in patients achieving disease clearance. The inclusion of disease clearance as a secondary endpoint provides an improved avenue of reducing disability by optimally controlling long-term disease complications.^36^ While a formal definition of disease clearance for CD lacks, this has important implications as achieving such a stringent outcome measure has been much debated. Furthermore, it has been suggested that disease clearance is best achieved with early therapeutic intervention.^36^ Our results suggest that combining biologic therapies may be a potential avenue for inducing disease clearance in patients with advanced complicated refractory disease. From the overall cohort of 21 CD patients, disease clearance was achieved in 24%. This is consistent with data from retrospective and post-hoc analyses of UC patients suggesting a potential attainment rate of up to 20%.^38,39^ Although dual biologics are gaining favor in the specialty community, insurance approval can be challenging. Overall, we have a high success rate with approvals for DBT due to the extreme refractory nature of cases with which we attempt DBT requests. The safety and efficacy of DBT delineated herein will provide insurance companies with the necessary evidence-based data to aid approval.

Inflammatory bowel disease with PSC is hypothesized to share a common underlying predisposition.^40^ The prevalence of PSC-CD ranges from 10% to 15%.^40^ Inflammatory bowel disease associated with PSC is said to have a characteristic phenotype.^40^ These subset of patients are also at a higher risk of colorectal cancer, colectomy, and overall mortality.^40^ From our cohort, only one patient had concomitant IBD-PSC. Despite optimized UST monotherapy and subsequent UST/VDZ dual therapy the patient failed to achieve disease control and was hospitalized for an acute severe flare of CD. Worsening of endoscopic disease with an SES-CD of 12 and HBI of 21 was noted. The patient eventually underwent an extended left hemi-colectomy. Our observations in this patient are consistent with the literature for severe disease and increased rates of colectomy in these subsets of patients. Current evidence also suggests no beneficial role of IBD-associated biologic therapies on PSC.^41^ The role of DBT has never been assessed in IBD-PSC patients before. Based on our experience, our patient did not gain any clinical benefit from concomitant UST/VDZ therapy. This study has several limitations. In our opinion, the most significant limitation includes the limited sample size and the biases associated with the retrospective design. Lack of patient randomization along with heterogeneity in baseline indication for initiation of DBT can be another concern. We believe an endoscopic assessment would have been a more reliable indicator of disease assessment in all cases. There were only 11 patients with follow-up endoscopy assessments. We report that a sufficient number of patients with follow-up endoscopies were not available in this study. Similarly, CTE readouts of more patients post-DBT would have better delineated transmural healing outcomes. Ideally, our aim was to assess efficacy, trends in disease status, safety signals, healthcare utilization, and therapeutic retention in refractory CD at baseline versus wk 52. This particularly limits our assessment of the sustainability of deep remission prior to and beyond the 52-wk timepoint. Long-term follow-up studies have formed the cornerstone of therapeutic safety and efficacy studies. We anticipate that some patients may also require a longer duration of combination therapy to achieve or lose response. Assessing trends in a 52 wk period should be the bare minimum to help inform guidelines regarding short-term efficacy and safety for dual biologic combinations. This will aid predictors of likely treatment failure and delayed response to improve therapeutic risk stratification. In addition to the management of refractory IBD and managing uncontrolled EIM, these predictors can be utilized to assess if a particular add-on therapy can be used as a temporary bridge and long-term maintenance and identify the optimal timing of future de-escalation to therapy. Based on this notion, characteristics of patients requiring the addition of other concomitant therapies can also be identified with possible triple therapy as a future therapeutic avenue in CD. Due to the limited cohort size significant predictors were not identified. Findings from a targeted timeline can be used to help determine the optimal timing and positioning of the second add-on therapy. More comprehensive patient-reported outcomes (PRO) such as CD-PRO could have a better-ascertained response to combination biologic therapy.

## Conclusion

Dual biologic therapy with UST/VDZ is well-tolerated and efficacious for refractory CD. This combination can be effectively used to control both active luminal disease and EIMs. It holds the potential to effectively induce disease clearance in complicated refractory CD patients. We identified no new or serious safety signals indicating good tolerability. This combination should be used with caution in patients with concomitant PSC. Further large-scale randomized trials are needed to assess the long-term efficacy and safety of this particular combination in a prospective manner.

## Supplementary Material

otae080_suppl_Supplementary_Figure_S1

otae080_suppl_Supplementary_Figure_S2

otae080_suppl_Supplementary_Figure_S3

otae080_suppl_Supplementary_Materials

## Data Availability

The data for this manuscript cannot be made available in accordance with the HIPAA rules. However, de-identified data may be made available upon reasonable request.
