# Supplementary figures and images for: Dual Biologic Therapy Induces Remission in Refractory Crohn’s Disease With Vedolizumab and Ustekinumab
Source: Crohns Colitis 360. 2024 Dec 17;7(1):otae080. doi: 10.1093/crocol/otae080 (PMC11759274; doi:10.1093/crocol/otae080)

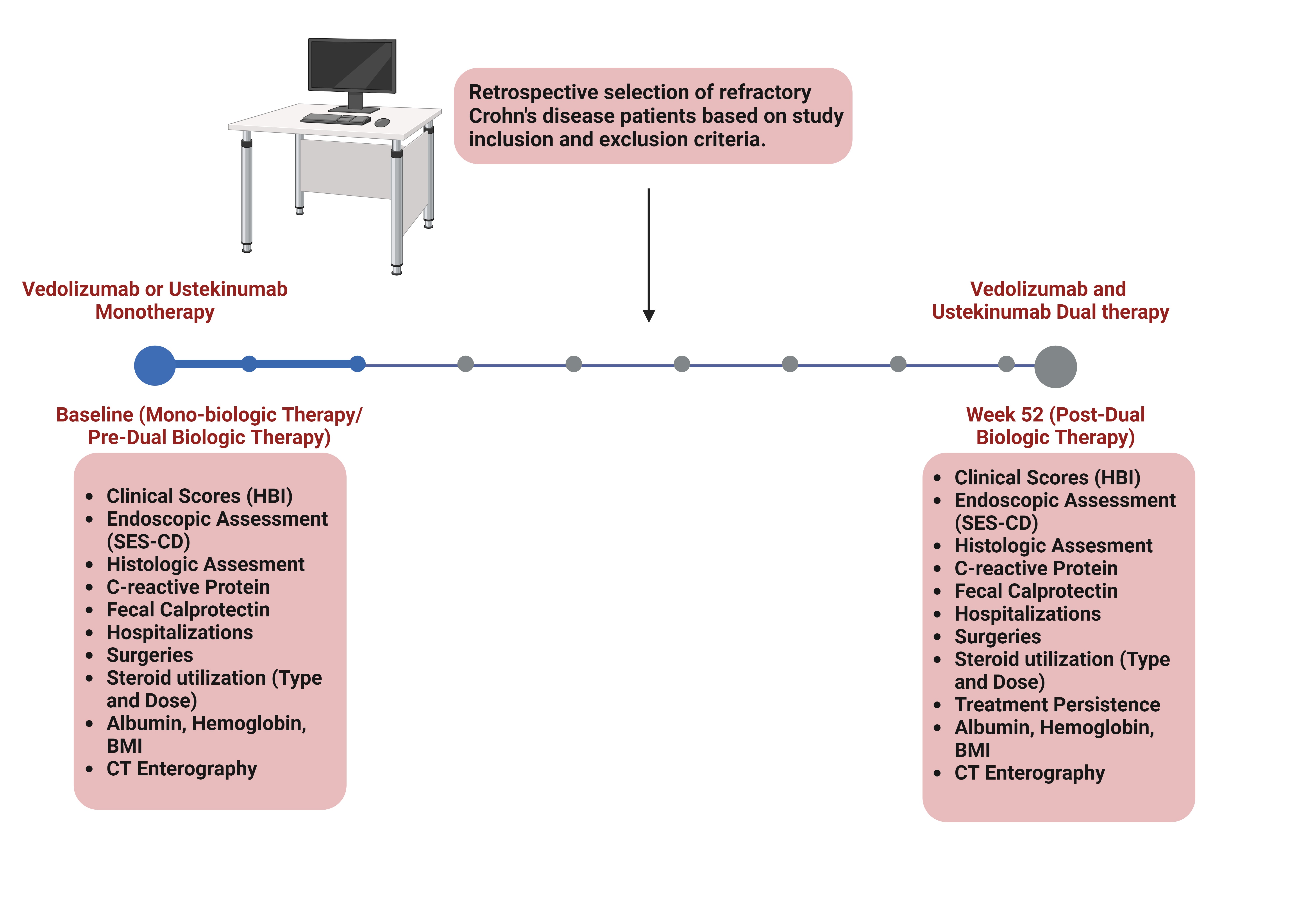

Supplement: otae080_suppl_Supplementary_Figure_S1 [file otae080_suppl_supplementary_figure_s1.jpeg]

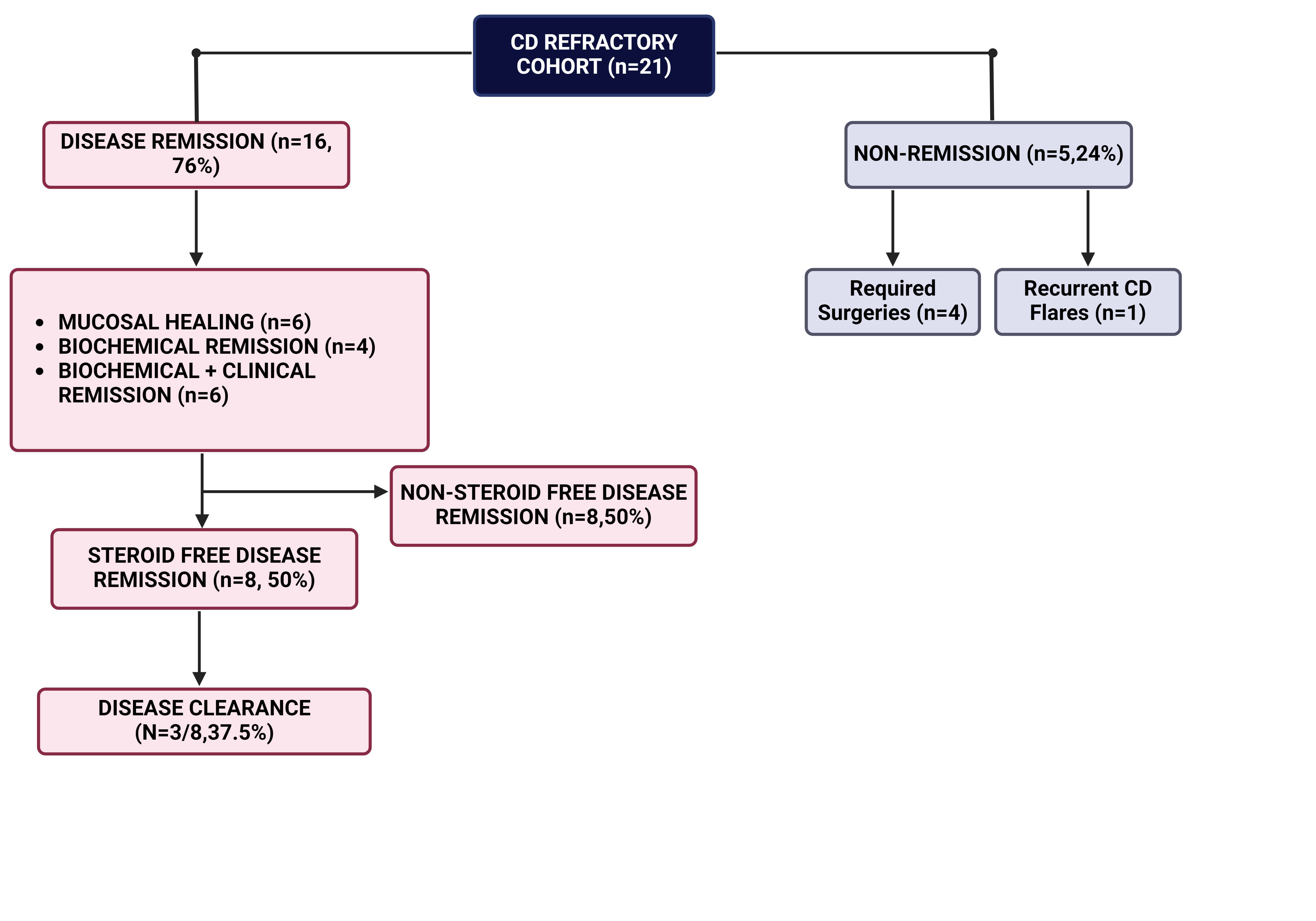

Supplement: otae080_suppl_Supplementary_Figure_S2 [file otae080_suppl_supplementary_figure_s2.jpeg]

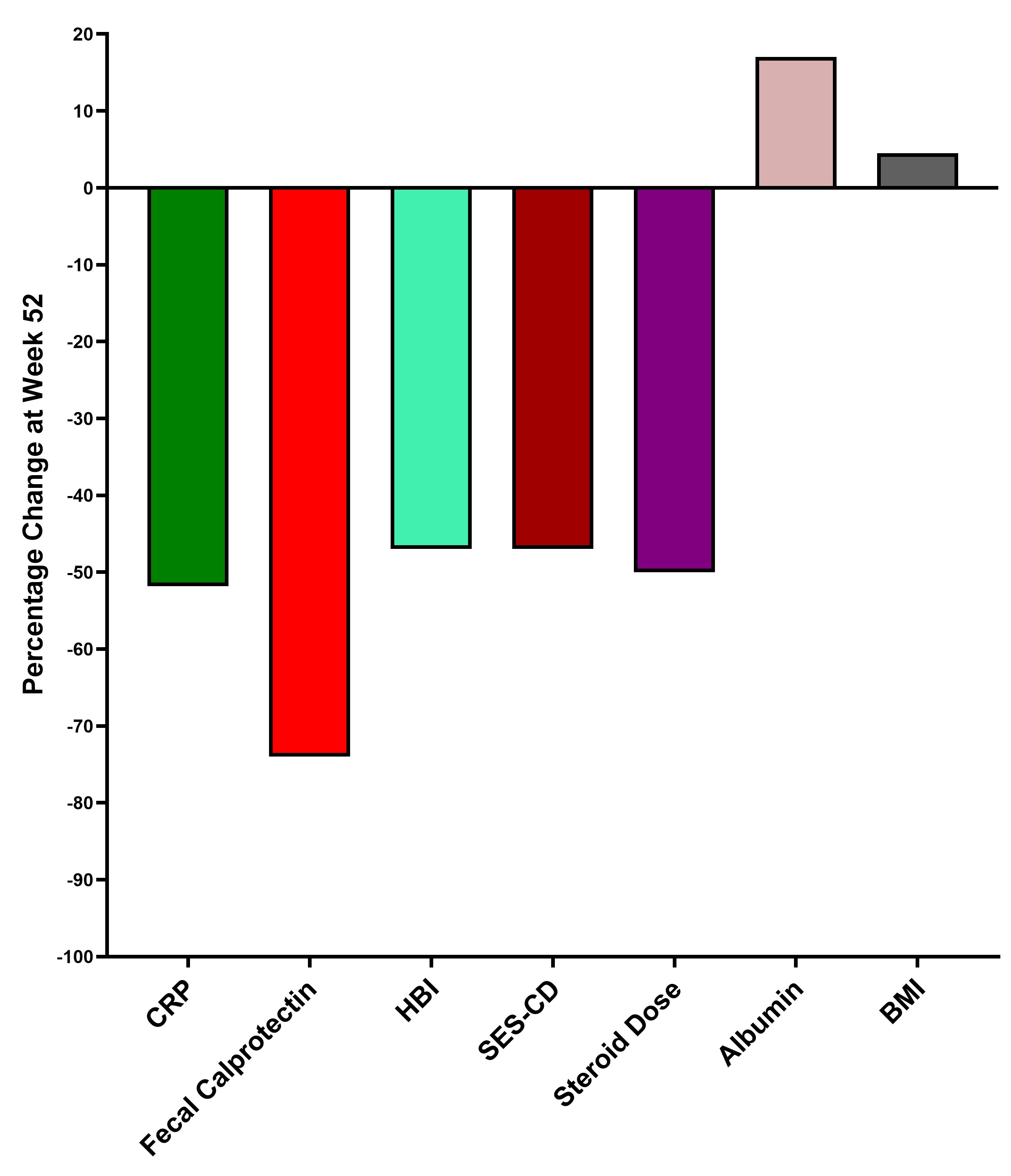

Supplement: otae080_suppl_Supplementary_Figure_S3 [file otae080_suppl_supplementary_figure_s3.jpeg]
