# Supplementary material for: Dual Biologic Therapy Induces Remission in Refractory Crohn’s Disease With Vedolizumab and Ustekinumab
Source: Crohns Colitis 360. 2024 Dec 17;7(1):otae080. doi: 10.1093/crocol/otae080 (PMC11759274; doi:10.1093/crocol/otae080)
Supplement: otae080_suppl_Supplementary_Materials [file otae080_suppl_supplementary_materials.docx]

**Supplementary Tables**

**Supplementary Table 1.** Summary of Radographic Features Pre-Dual Biologic Therapy on Computed Tomography Enterography.Per Unique Patient

| **Radiographic Feature** | **Overall Pre-Dual Biologic Therapy (n=17)** |
| --- | --- |
| **Bowel Wall Thickness (> 3 mm)** | 17 (100%) |
| **Hyperenhancement** | 17 (100%) |
| **Comb Sign** | 9 (53%) |
| **Active Inflammation** | 17 (100%) |
| **Peri-enteric Inflammation** | 14 (82%) |
| **Abscess** | 0 |
| **Fistulas** | 4 (24%) |
| **Strictures** | 4 (24%) |

***All features are represented in unique patients.**

**Supplementary Table 2.** Comparison of Paired Pre- and Post- Dual Biologic Therapy Radographic Features on Computed Tomography Enterography.

| **Radiographic Feature** | **Pre-Dual Biologic Therapy (n=11)** | **Post-Dual Biologic Therapy (n=11)** |
| --- | --- | --- |
| **Bowel Wall Thickness (> 3 mm)** | 11 (100%) | 5 (45%) * |
| **Hyperenhancement** | 11 (100%) | 5 (45%) * |
| **Comb Sign** | 6 (45%) | 1 (9%) * |
| **Active Inflammation** | 11 (100%) | 5 (45%) * |
| **Peri-enteric Inflammation** | 9 (81%) | 4 (36%) * |
| **Abscess** | 0 | 0 |
| **Fistulas** | 4 (36%) | 2 (18%) * |
| **Strictures** | 2 (18%) | 1 (9%) * |

***Represents radiographic findings from CTE in 5 non-responders to dual biologic therapy. All features are representative of unique patients.**

**Supplementary Figure Legend:**

**Supplemental Figure 1:** Assessment summary of clinical outcomes and healthcare utilization characteristics on ustekinumab or vedolizumab monotherapy versus dual ustekinumab and vedolizumab therapy.

**Supplemental Figure 2:** Summary of outcome measures achieved with dual ustekinumab and vedolizumab therapy at week 52.

**Supplemental Figure 3:** Pre-post therapy percentage change in c-reactive protein, fecal calprotectin, Harvey Bradshaw index, simple endoscopic score, daily steroid dose, albumin and body mass index.
